# Morphology analysis of PEEK 450G using scanning electron microscopy directly on fast scanning calorimetry chips

**DOI:** 10.1038/s41598-024-69164-2

**Published:** 2024-08-08

**Authors:** Cleiton André Comelli, Nan Yi, HenkJan van der Pol, Oana Ghita

**Affiliations:** 1 Faculty of Environment, Science and Economy, University of Exeter, Harrison Building, Streatham Campus, North Park Road, Exeter, EX4 4QF UK; 2 BOND 3D, Institutenweg 50, 7521 PK Enschede, The Netherlands

**Keywords:** PEEK, Additive manufacturing, FSC, Crystallisation, Mechanical engineering, Polymers

## Abstract

To explore the morphology of polyetheretherketone (PEEK), this study employed fast scanning calorimetry (FSC) and scanning electron microscopy (SEM). The objective was to observe the PEEK microstructure under various thermal profiles replicating the additive manufacturing material extrusion process. Samples were observed using SEM directly from the FSC chips, allowing high-accuracy evaluation of the microstructure relative to the thermal profiles. This approach allowed for the evaluation of the microstructure with high accuracy concerning the thermal profiles to which the samples were previously exposed. Each sample was coated with a 10 nm layer of gold–palladium (20–80% ratio), and no etching was necessary to observe the micro features of the microstructure. The approach enabled successful observation and quantification of PEEK microstructure, linking substrate temperature and temperature peaks to microstructural outcomes. Notably, temperature peaks during the process enhanced the formation of well-developed, thick lamellae due to increased chain mobility. Additionally, embryos formed post-remelting of the substrate structure were observed.

## Introduction

Crystallisation is a kinetic process where molecular chains or atoms transition from a disordered state to an ordered arrangement of greater stability. It can be described as a phase transformation driven by excess free energy in the system. Crystallisation kinetics, influenced by temperature changes, can be isothermal, non-isothermal with constant cooling rates, or non-isothermal with varying cooling rates, common in manufacturing processes^1–3^.

Under isothermal conditions, the crystallisation rate of a polymer varies with temperature, following a bell-shaped function. Therefore, there is an optimal temperature at which the crystal formation rate is maximal, decreasing at higher and lower temperatures from the optimal temperature^1^. For constant cooling conditions, the cooling rate impacts nuclei activation, with higher cooling rates leading to crystallisation at lower temperatures, whereas lower cooling rates increase the crystallisation temperature^4,5^.

In polymers, crystallisation results in a microstructure that organizes polymeric chains into a crystalline lattice composed of unit cells. This structure contrasts with the amorphous microstructure, characterized by randomly arranged polymer chains, and the semi-crystalline microstructure, which comprises both amorphous and crystalline regions^2,6,7^. Understanding these microstructural differences is crucial for predicting the mechanical properties and performance of polymeric materials in various applications^8,9^.

Crystallisation occurs within a temperature range from the glass transition (Tg) to the melting temperature (Tm) and is not instantaneous^10^. Typically, the process creates a multi-layered, chain-folded structure with both crystalline and amorphous regions. The polymer chains arrange themselves with adjacent crystals to form structures called spherulites^2,3,11^.

The shape of spherulites is influenced by crystallisation conditions, the nature of crystallisation, and the chemical properties of the polymer. Polymers with linear chains or polar groups that facilitate strong intermolecular or hydrogen bonds crystallize more readily, while closely spaced or asymmetric branches or side groups can hinder polymer crystallisation^2,11–14^.

The kinetics of crystallisation in PEEK 450G, a semi-crystalline polymer, is influenced by factors such as cooling rate, nucleation density, and molecular weight. Understanding its crystallisation behaviour is essential for comprehending the material's properties and performance. Variations in PEEK 450G microstructure can arise due to differences in crystallinity levels or processing methods, which are affected by mechanical stresses or thermal profiles^11,12,14–16^.

Wang et al.^16^ demonstrated that quench crystallization of PEEK thin films produced novel fibre-like crystal structures with higher elongation at break compared to traditional spherulitic crystal structures formed through melt crystallization. Microscopic fracture analysis revealed that the quenched films exhibited extended strain and ductility, highlighting the impact of crystal morphology on the mechanical performance of PEEK^16^. Comelli et al.^15^ found that drawn PEEK feedstock filament exhibited significantly higher mechanical strength and distinct XRD spectra, with greater crystallinity in filaments extruded from drawn feedstock, emphasizing the melt-memory effect.

Different variations of PEEK can also present different behaviours, as shown by Chu and Schultz^17^. The authors investigated the effect of spherulite size on fracture by comparing the structures of PEEK 150P and PEEK 450G. Using SEM for further observation of the spherulites through polishing and permanganic etching, they concluded that PEEK 150P had larger spherulites compared to PEEK 450G due to a higher nucleation density in the polymer with higher molecular weight. They attributed this effect to the impingement of spherulites, preventing growth, similar to what was observed in the material with lower molecular weight. Additionally, the variation in spherulite size was influenced by the processing temperature, with higher temperatures resulting in larger spherulites. The authors also noted that the size of the spherulites increased with the melting temperature^17^.

Since temperature significantly influences the morphology of spherulites, different thermal processing profiles with varying temperatures are also expected to impact the resulting microstructure. Research has established connections between thermal history and critical factors such as spherulite size, number of melting peaks, nucleation density, time, temperature, and molecular weight^10,18–20^.

Basset et al.^10^ explored the lamellar morphology of PEEK using thermal analysis and scanning electron microscopy (SEM), along with permanganic etching. They observed that the structure of the spherulites in PEEK consists of a chain of main branches with subsequent filling between them. The authors observed double melting peaks (commonly observed in PEEK thermal analysis) and correlated them with these two structures, with the peak of higher melting temperature being related to the main branches of the spherulites, while the peak with a lower melting temperature was related to secondary crystalline structures growing between these branches^10^.

Tan et al.^20^ examined the crystallization kinetics of PEEK from its metastable melt using differential scanning calorimetry (DSC). During the heating scan of semicrystalline PEEK, they observed a double endothermic behaviour. This phenomenon was attributed to the melting of thinner lamellar crystal populations, which then recrystallized into slightly thicker lamellae. Transmission electron microscopy (TEM) and wide-angle X-ray diffraction (WAXD) confirmed the formation of lamellar crystals with varying thermodynamic stability during isothermal crystallization. Only the more stable crystals remained after partial melting^20^.

Vaes et al.^21^ investigated the development of crystallinity in semi-crystalline polymers used in fused filament fabrication (FFF). They recorded the thermal profile during the printing of single-layer walls of two PA 6/66 copolymers with different molecular weights using Infrared Thermography. By varying print settings, they assessed their impact on crystallinity. The study found that liquefier temperature and print speed had minimal influence, while the build plate temperature, when above the polymer’s glass transition temperature, had significant annealing effects. Polymers with lower molecular weights exhibited higher crystallinity. Fast scanning chip calorimetry (FSC) was used to effectively simulate thermal cycles, proving useful in predicting part crystallinity and optimizing FFF processes^21^.

In a recent study, Lee et al.^22^ examined how convection within a commercial 3D printer (Funmat HT—Intamsys) impacts the morphology and thermal history of PEEK during additive manufacturing. Utilizing differential scanning calorimetry (DSC) and polarized optical microscopy (POM), they assessed the crystallinity of PEEK (3DXTECH) based on its thermal history, emphasizing the role of cooling. Their findings indicated that the temperature history during the process significantly influenced PEEK's microstructure. Samples subjected to higher cooling rates exhibited pronounced cold crystallization, whereas slower cooling rates produced larger spherulites, ranging from 15 to 30 μm at a cooling rate of 20 °C per minute. Conversely, extremely rapid cooling rates (over 1000 °C per minute) resulted in spherulitic structures too small to be detected by POM^22^.

These findings showcase the critical role of thermal management in manufacturing processes and its impact on the final microstructure and properties of printed parts. Also, examining the resulting microstructure in PEEK parts produced through additive manufacturing processes is of particular interest, especially considering the unique thermal profiles involved in the technique. However, optical microscopy techniques have limitations in observing spherulites due to rapid cooling and sudden temperature variations during processing, as demonstrated by Lee et al.^22^. Therefore, alternative methods such as scanning electron microscopy (SEM) or transmission electron microscopy (TEM) have been employed to overcome these limitations.

In this context, this study introduces a novel methodology for investigating the morphology of PEEK under complex thermal profiles by combining FSC and SEM techniques. This integration offers several advantages for analysing the morphology of PEEK under such profiles. Firstly, the utilisation of FSC enables precise replication and control over the simulated thermal profiles, allowing the process to be frozen at any desired time. This capability is particularly valuable as it facilitates capturing the material’s morphology at specific stages of the simulated thermal cycle. Moreover, evaluating the sample directly within the FSC chip enables the acquisition of high-resolution images of the morphology without the need for surface etching, thereby contributing to a more detailed analysis of the microstructural features.

## Materials and methods

### Raw materials

Victrex PEEK 450G was used for this study. The material was supplied by BOND 3D in rod shaped samples and small particles were obtained from the centre part of the rod. The main characteristics of this material are presented in Table 1.

**Table 1:** PEEK 450G main properties^23^.

Property	Typical value
Tensile strength (MPa)	98
Tensile elongation (%)	45
Tensile modulus (GPa)	4
Melting point (°C)	343
Glass transition temperature Tg (°C)	143
Density (g cm^−3^)	1.3
Drying temperature/time	150 °C/3 h or 120 °C/5 h (Residual moisture < 0.02%)

### Replication of simulated thermal profiles from MEX via FSC and microstructure analysis using SEM techniques

The core methodology of this study involves replicating the simulated thermal profiles from a material extrusion (MEX) printing process within a fast scanning calorimetry (FSC) analysis framework. FSC allows the use of a very small sample placed in a chip which can be precisely exposed to any investigated thermal profile and represents the specific interest points from a real print job. This innovative approach allows for the interruption of the replicated process at any predetermined point along the simulated thermal profile, thereby enabling an in-depth evaluation of the resulting microstructure. The microstructure obtained through this method is anticipated to closely resemble that of the actual MEX printing process. Scanning electron microscopy (SEM) was employed directly on the FSC samples to assess the microstructure, thus eliminating the need for sample cutting or additional handling.

### MEX thermal profile simulation

**Figure 1:**
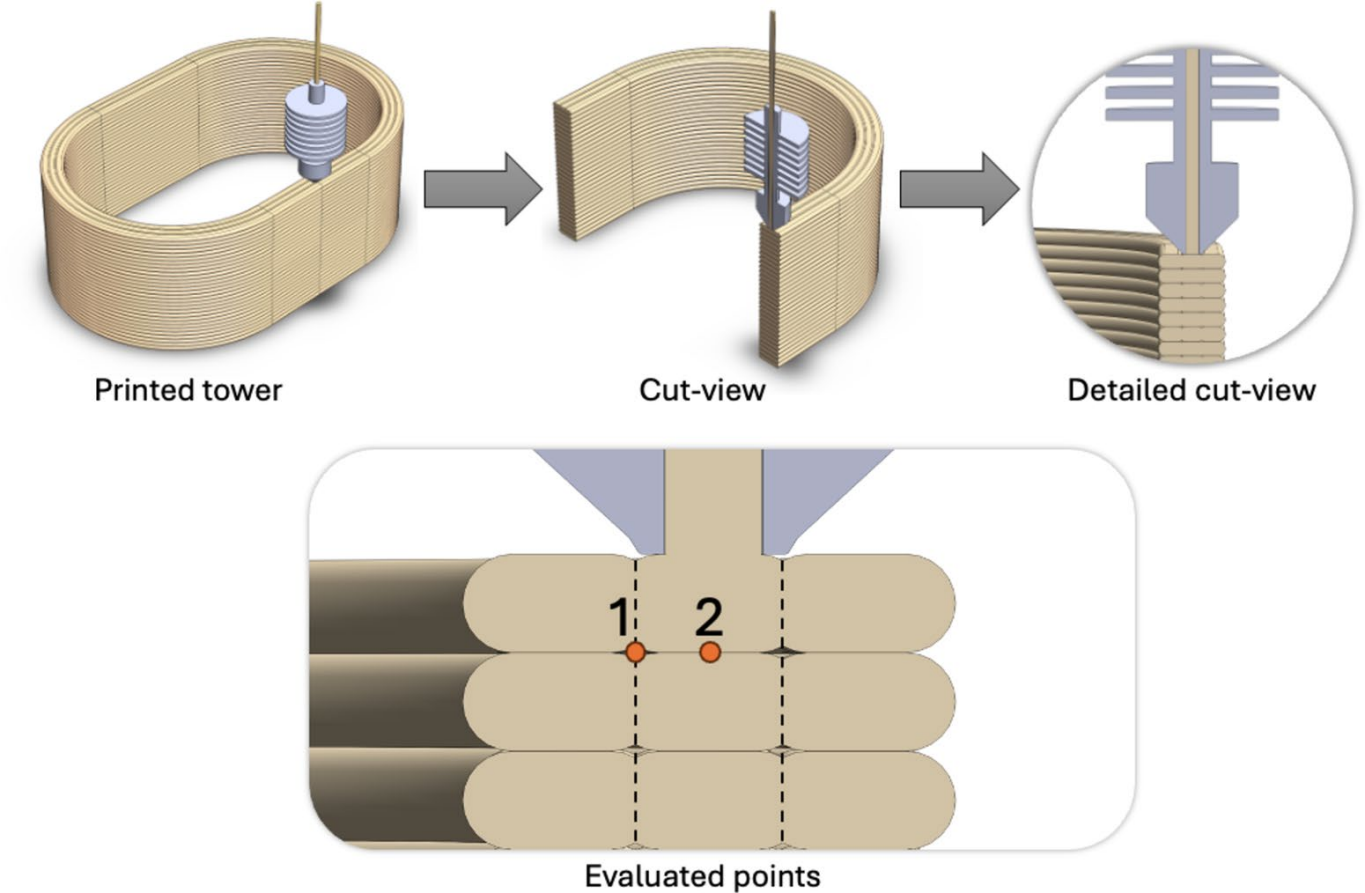
Basic building pattern of the 3-layer thick wall and the simulated nodes used for thermal analysis. The section view shows node 1 (edge node) and 2 (central node), which were selected for the experiments since they correspond to points with direct thermal influence from two tracks (point 2) and 4 tracks (point 1)^24^.

The MEX process simulated thermal profiles were provided by BOND 3D and represent the temperature as a function of time at specific points of a cross-section of a three-filament thick wall tower. Four thermal profiles were generated, using two nodes (node 1 and node 2) and two levels of substrate temperature for each node. Figure 1 shows a section view of the simulated printed wall and the specific points evaluated.

**Figure 2:**
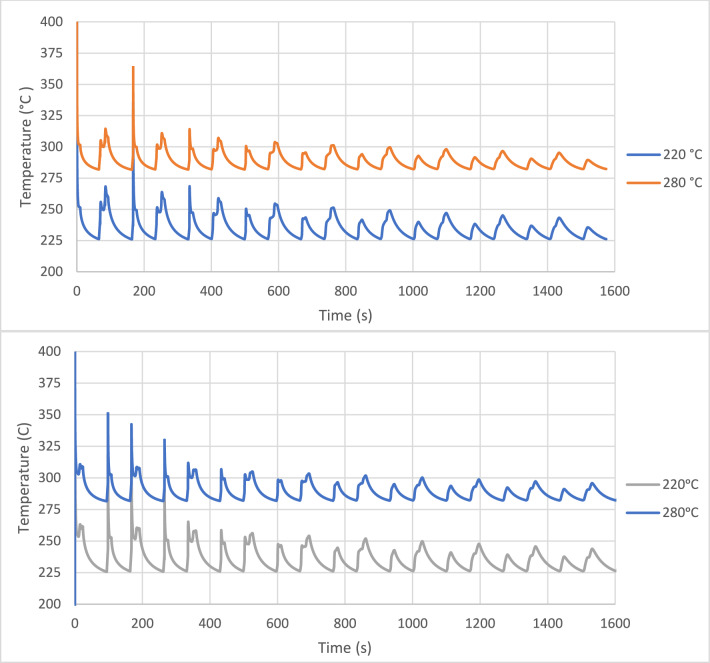
Top chart: Thermal profiles for node 2 for substrate temperatures of 220 °C and 280 °C and a return time of 167 s. Bottom chart: Thermal profiles for node 1 for substrate temperatures of 220 °C and 280 °C and a return time of 97 s^24^.

Initially, node 2 was evaluated, the goal was to understand the substrate temperature influence on the microstructure after the entire process simulation, therefore the two thermal profiles used to prepare the samples corresponded to a substrate temperature of 220 °C and 280 °C with a return time of 167 s. After the entire simulation of the thermal profiles, each sample was quenched to 30 °C (@-4000 °C s^−1^). In a second step, the experiment was repeated, this time for the node 1. Again, the two substrate temperature levels (220 °C and 280 °C) were simulated. The choice of these two thermal profile patterns was motivated by the significant difference observed in the melting curves for both situations, when the material was analysed by FSC. Also, this time the tested return time was 97 s. The selected thermal profiles for nodes 2 and 1 are shown in Fig. 2^24^.

Since the complex variation of temperatures could not be directly input in the FSC, each thermal profile was simplified using linear approximation, this was accomplished using 84 straight segments (85 points). 43 key points were selected to measure the instant crystallinity, always using a quenching (@-4000 °C s^−1^) and melting cycle (@1000 °C s^−1^) for both, nodes 1 and 2.

**Figure 3:**
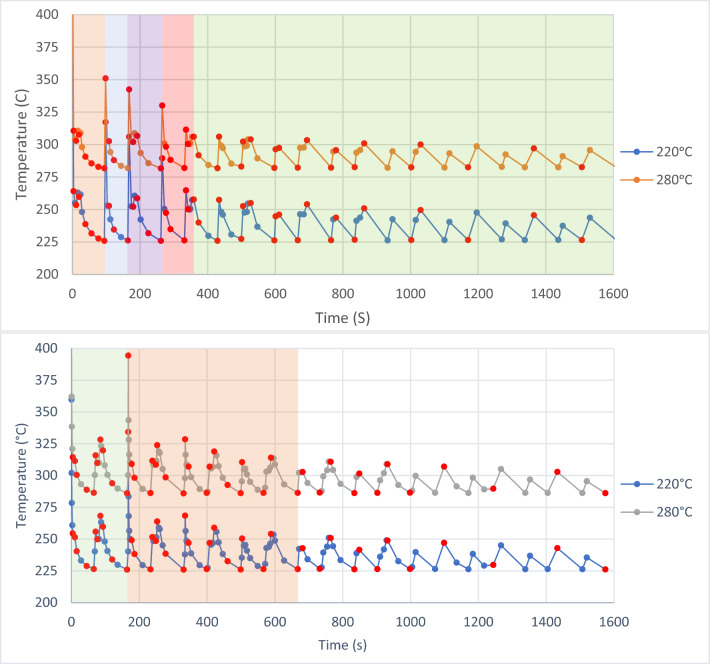
Linear approximation of the BOND 3D thermal profiles for node 1 (top chart) and node 2 (bottom chart)^24^.

The crystallinity was always assessed with the same heating rate for all points, in this case, 1000 °C s^−1^, which may be different when compared with real process heating rates, however, this procedure allowed to understand and compare the crystallinity development and evaluate the resulting melting curves over the entire thermal profile. The linear approximation for nodes 1 and 2 is shown in Fig. 3. The red dots are the points selected to measure the crystallinity and the colours are included in the different sections to facilitate the interpretation presented in specific charts in the results section.

In this study, we evaluated the resulting morphology for each temperature, and understanding the evolution of crystallinity helps in evaluating the observed morphologies.

One additional point was also simulated to observe the remelting effect. For this, the thermal profile corresponding to node 1 was selected. In this case, the substrate temperature tested was 220 °C, and the process was interrupted right at the peak formed during the following filament deposition.

### Fast scanning calorimetry

FSC is a relatively new thermal analysis technique that offers some advantages over traditional DSC. It provides rapid heating and cooling rates, allowing for the analysis of crystallisation in fast-crystallizing polymers, such as PEEK 450G, within temperature ranges where crystallisation rates are maximal. In this context, the technique was used for a detailed study of the crystallisation of the material under MEX process conditions^24^.

The methodology involved running each thermal profile up to the desired points where crystallinity was to be assessed. Upon reaching the target point, a rapid quench to room temperature followed by a fast-heating cycle was performed. This approach enabled the measurement of the specific crystallinity at each designated point. For the points where morphology was to be evaluated, only the quenching was carried out to preserve the microstructure at that specific point^24,25^.

SEM sample preparation was also conducted using this technique, with the FSC chips themselves serving as SEM samples after the necessary coating step. The ceramic UFS1-type sensors were used. They include a total of 16 thermocouples—8 for the sample and 8 for the reference—symmetrically placed around the sample and reference areas. The sensor specifications include heating rates from 0.1 to 50,000 °C s^−1^ and cooling rates from 0.1 to 4000 °C s^−1^, with a recommended sample mass for polymers ranging from 5 to 400 ng.

The samples used weighed between 77 and 265 ng and were obtained from small particles removed from the centre portion of the PEEK rods using a scalpel. The mass was calculated using the superposition of the FSC and DSC scanning rates, generating samples with similar crystallinity (since the same cooling rate was applied to both DSC and FSC samples)^26^. In this method, if the crystallinity is similar, by definition, both specific enthalpies of melting should be equal, and the mass of the sample can be estimated by the following equation.

1 $$ m,FSC = \frac{{\Delta H_{m,FSC} }}{{\Delta H_{m,DSC} }}*m,DSC $$

where $\Delta H_{m,FSC}$ and $\Delta H_{m,DSC}$ are the melting enthalpies, measured by the FSC and DSC, respectively, and $m,FSC$ and $m,DSC$ are the sample masses^24^.

### Scanning electron microscopy (SEM)

To evaluate the samples, A Zeiss Gemini SEM 500 was used. The equipment enables imaging at sub-nanometre, with a resolution up to 0.8 nm at 1 kV, an accelerating voltage range from 0.02 to 30 kV and a magnification range from × 50 to × 2.000.000. The main goal of the experiment was to observe the crystalline phase at the end of a simulated MEX process using the FSC. However, since the FSC was also used to prepare the samples, it provided the opportunity to observe specific microstructures for different process configurations and durations.

**Figure 4:**
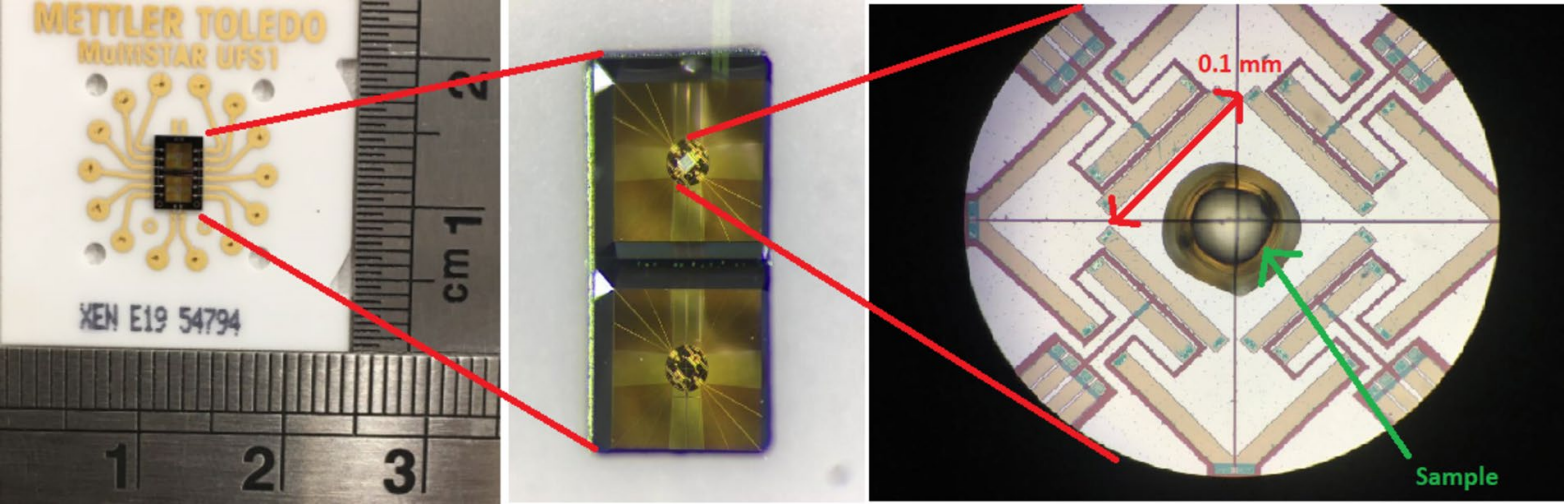
SEM sample: a PEEK sample on a FSC chip^24^.

Initially, there was a challenge in removing the FSC sample from the fragile metallic membrane on the chip, rendering it impractical. To address this issue, scanning electron microscopy (SEM) was employed to directly observe the PEEK sample within the FSC chip. However, due to the coating procedure necessary for SEM analysis, the chip could not be reused in the FSC. As a result, a new chip was required for each tested thermal profile. The PEEK sample in the FSC typically had a thickness ranging from 10 to 50 µm, and a thin coating of 10 nm gold/palladium (20/80 ratio) was applied prior to conducting SEM analysis. It is important to note that etching was not needed in this process. The sensor with one sample inserted is shown in Fig. 4.

Since a new chip was required for each analysis, a limited number of points had to be selected from the thermal profiles. As a result, a total of five samples were prepared, corresponding to high and low substrate temperatures for each of the tested nodes. Additionally, a fifth sample was used to observe the microstructure during the heat peak produced by the subsequent layer deposition.

### Spherulite morphology analysis

A method for analysing SEM images has been developed to quantitively assess the morphology of observed spherulites. This method involves several steps. First, OTSU thresholding was applied to separate crystalline regions and amorphous regions. Then, a matrix of 10,000 evenly distributed points was generated across the segmented image. Next, points that fall outside the crystalline regions were excluded. The lamellar widths of the crystalline regions were measured at the remaining points. Finally, the histograms of the recorded lamellar widths were plotted to illustrate the distribution.

## Results

### Crystallinity evolution and resulting melting traces for node 1 and 2

#### Crystallinity and melting curves analysis on node 2

**Figure 5:**
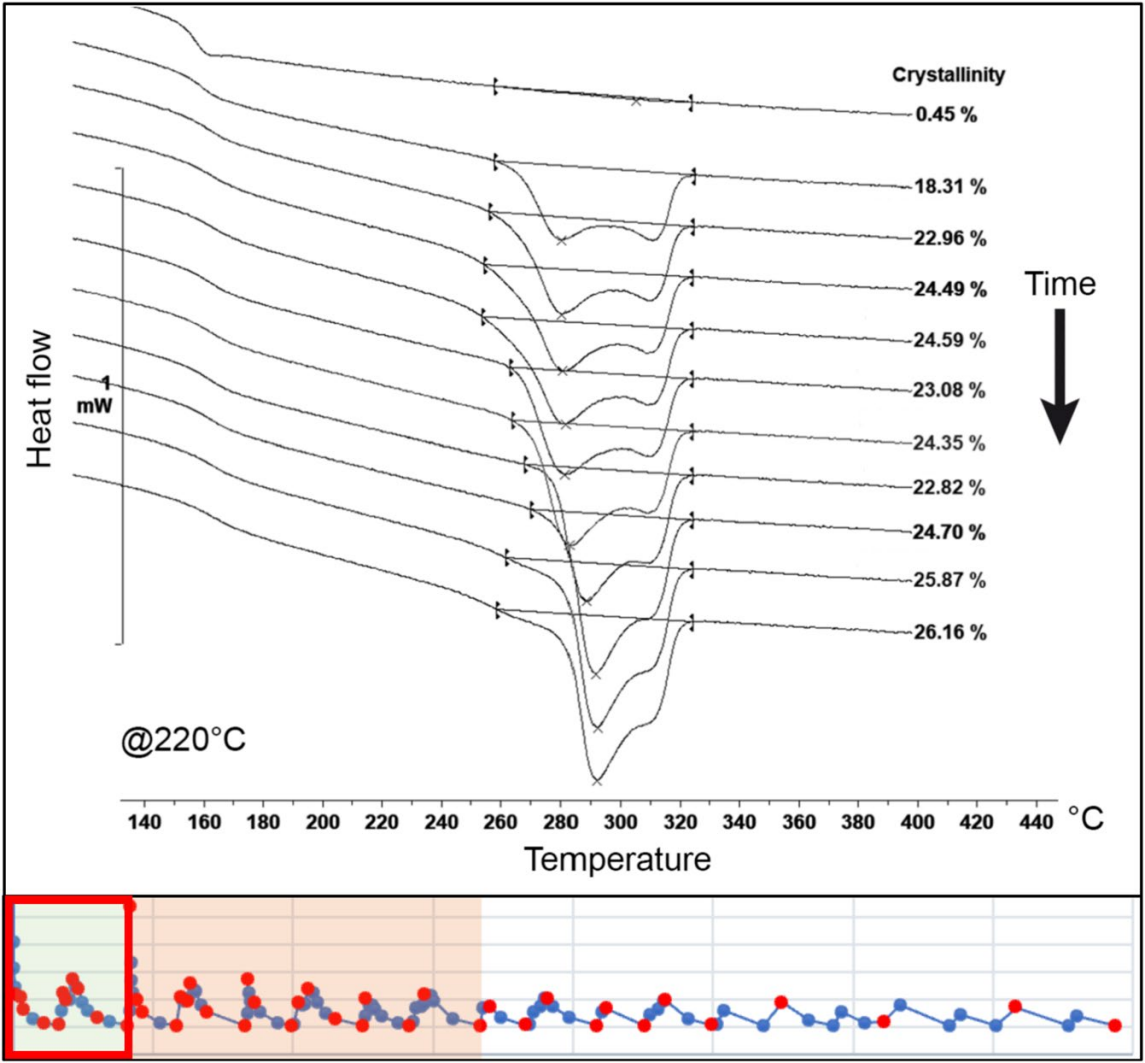
Evolution of the crystallinity before the second layer deposition on node 2 at 220 °C^24^.

**Figure 6:**
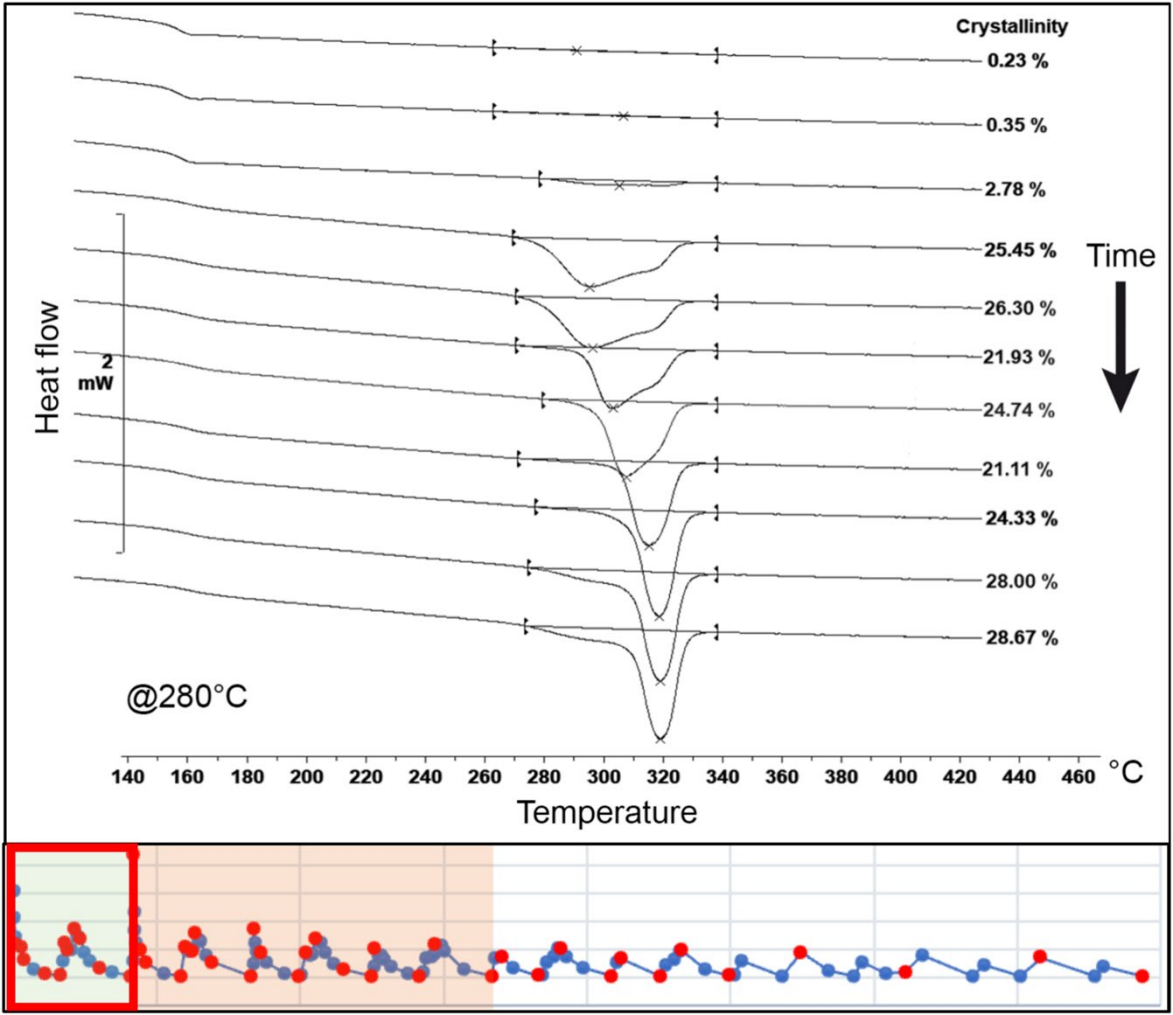
Evolution of the crystallinity before the second layer deposition on node 2 at 280 °C^24^.

The melting curves and crystallinity were evaluated across the entire thermal profile. The evolution of crystallinity during the initial stage of the process, from the moment when the PEEK exits the extrusion nozzle to the point just before the deposition of the subsequent layer, is depicted in Figs. 5 and 6 for temperatures of 220 °C and 280 °C, the small diagram on the bottom shows the correspondent process segment highlighted by the red square.

At a temperature of 220 °C, one can observe a rapid initial growth of the crystalline portion of the material, quickly reaching levels close to 20% and 26% at the critical point, just before the deposition of the second layer. At higher temperatures, crystal growth is slower initially, but it reaches 28% crystallinity at the critical point, as illustrated in Fig. 6.

For lower temperatures, the polymer exhibits lower melting temperature peaks, indicating reduced reorganization of the polymer chains and faster crystallisation rates. This results in crystallites impingement and decreased mobility.

In contrast, higher oven temperatures promote greater reorganization of crystals, as evidenced by the higher melting point of the resulting peaks. Additionally, the low-temperature melting peak shifts towards the high-temperature peak until they merge into one peak (prior to the deposition of the second layer).

**Figure 7:**
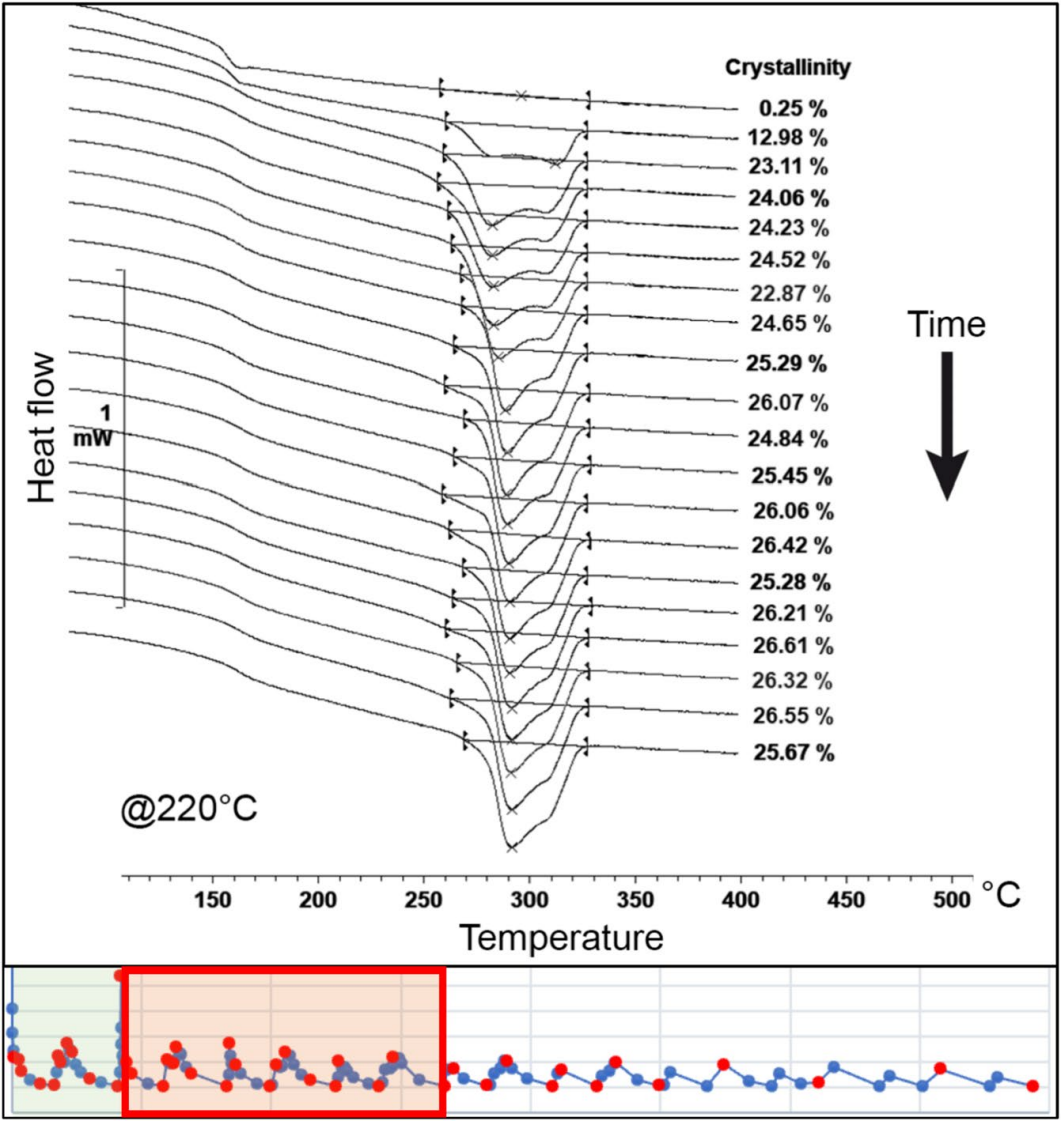
Evolution of the crystallinity after the second layer deposition on node 2 at 220 °C^24^.

**Figure 8:**
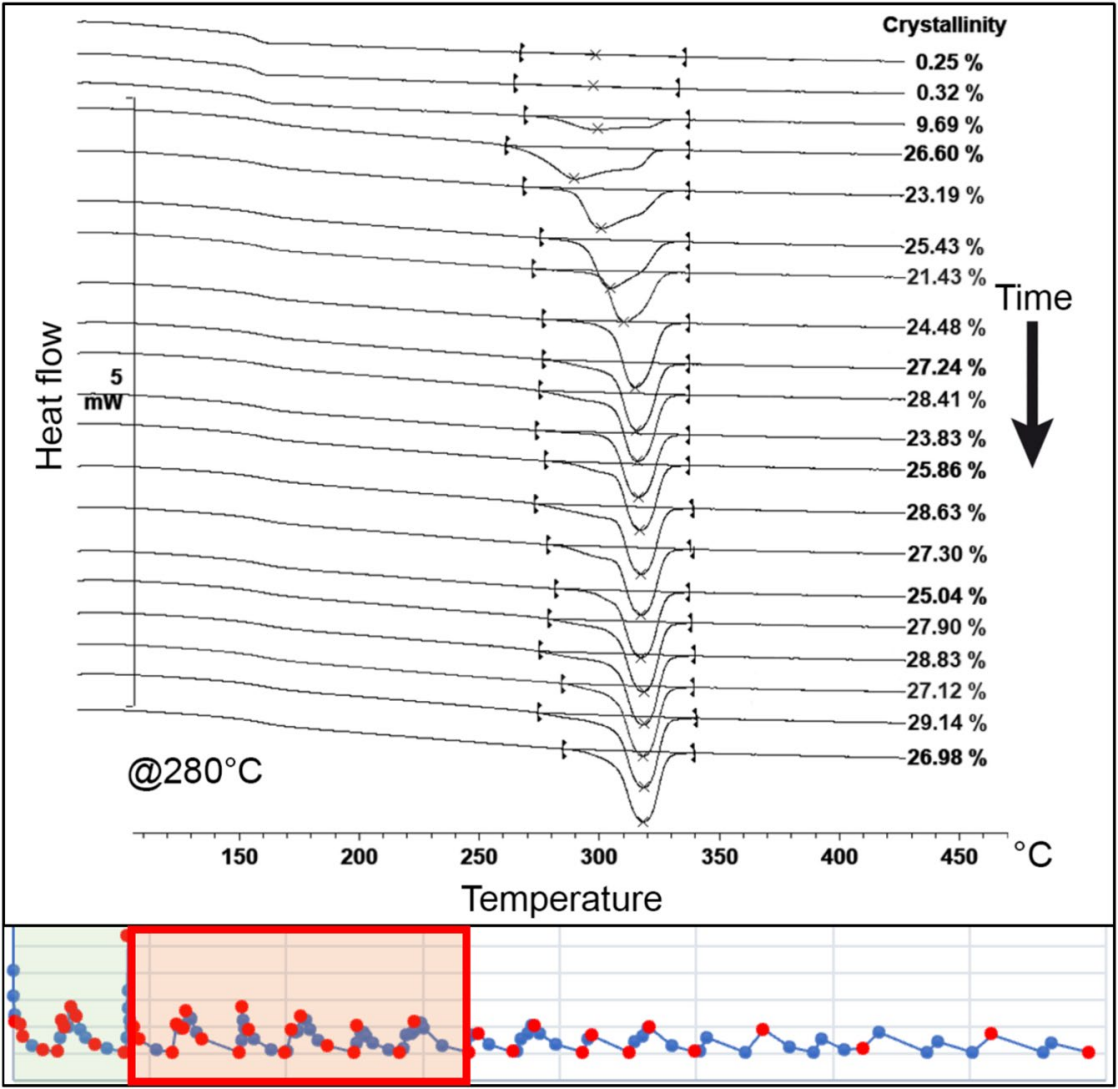
Evolution of the crystallinity after the second layer deposition on node 2 at 280 °C^24^.

The second evaluated region corresponds to the points after the deposition of the subsequent layer. Here, it is evident that crystallinity experiences a significant reduction, gradually increasing thereafter. This observation is consistent with the presence of more widely spaced melting peaks for lower temperatures and closer peaks for higher temperatures. The evolution of the melting peaks for both tested temperatures is depicted in Figs. 7 and 8.

At lower temperatures, crystallisation begins earlier after the deposition of the second layer. The growing structure exhibits two well-defined melting peaks, with a prevalence of the low-temperature peak, which gradually shifts towards the primary peak with a higher melting point.

In contrast, at higher temperatures, the onset of crystallisation is delayed. Initially, the structure displays a prevalence of the lower melting peak; however, it rapidly shifts towards the primary peak, resulting in a higher melting point.

**Figure 9:**
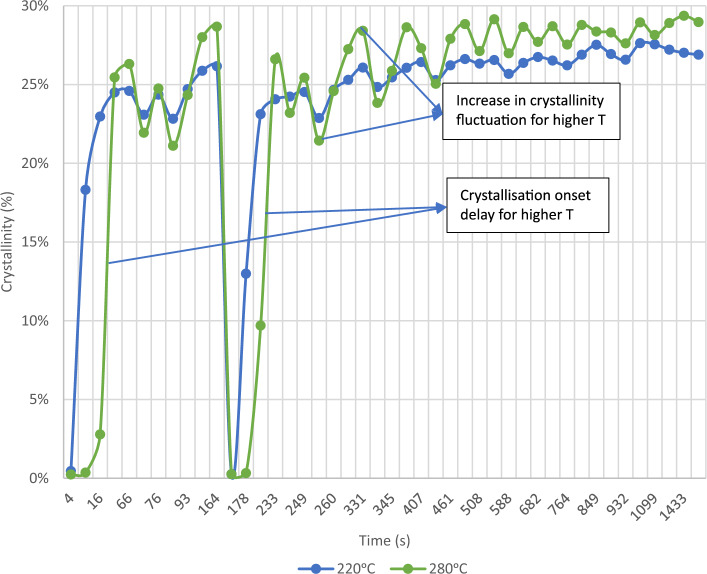
Crystallinity evolution within the MEX process on node 2 for 220 °C and 280 °C^24^.

The analysis of crystallinity also revealed significant differences between the tested temperatures. As the temperature increased, there was a notable increase in the fluctuation of the measured values, which can be attributed to the enhanced mobility of the polymeric chains under these conditions. This phenomenon correlates with the observed higher melting temperatures and increased levels of crystallinity. Moreover, higher temperatures led to a delay in the onset of crystallisation. The evolution of crystallinity for the tested temperatures is illustrated in Fig. 9.

#### Crystallinity and melting curves analysis on node 1

**Figure 10:**
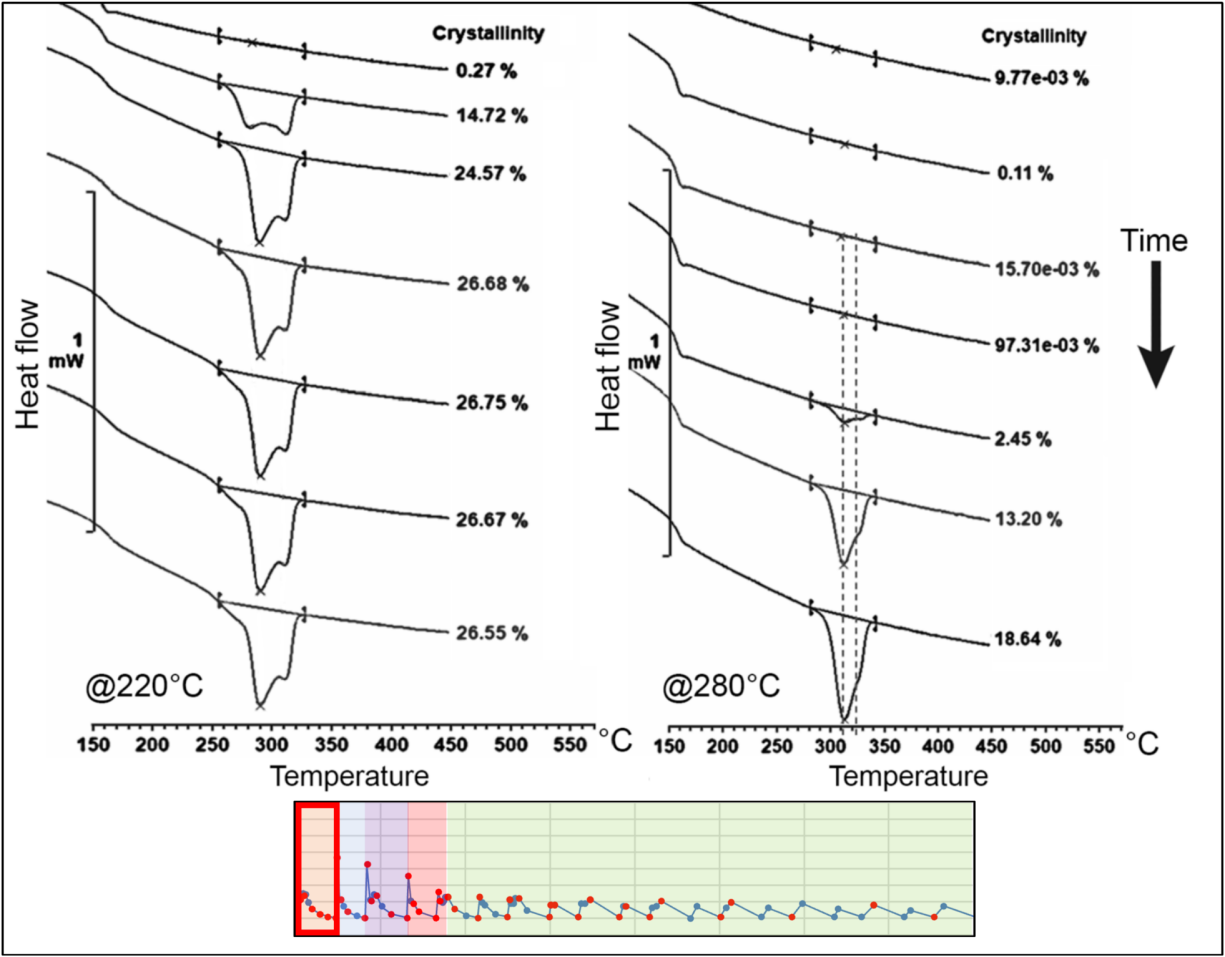
Evolution of the crystallinity before the first heat peak (region 1) on node 1 at 220 °C and 280 °C^24^.

For node 1, the thermal cycle was divided into six regions to better understand the influence of each heat input from the adjacent filaments on the evolution of crystallinity. In the first region, immediately after the material extrusion, there was a rapid growth of the crystalline phase. At a temperature of 220 °C, the absolute crystallinity reached a peak of approximately 26% before remelting occurred due to the first heat peak. In contrast, at 280 °C, a delay in the onset of crystallisation was observed, resulting in a lower crystallinity level of approximately 18%. The difference between the temperatures of 220 °C and 280 °C is depicted in Fig. 10.

**Figure 11:**
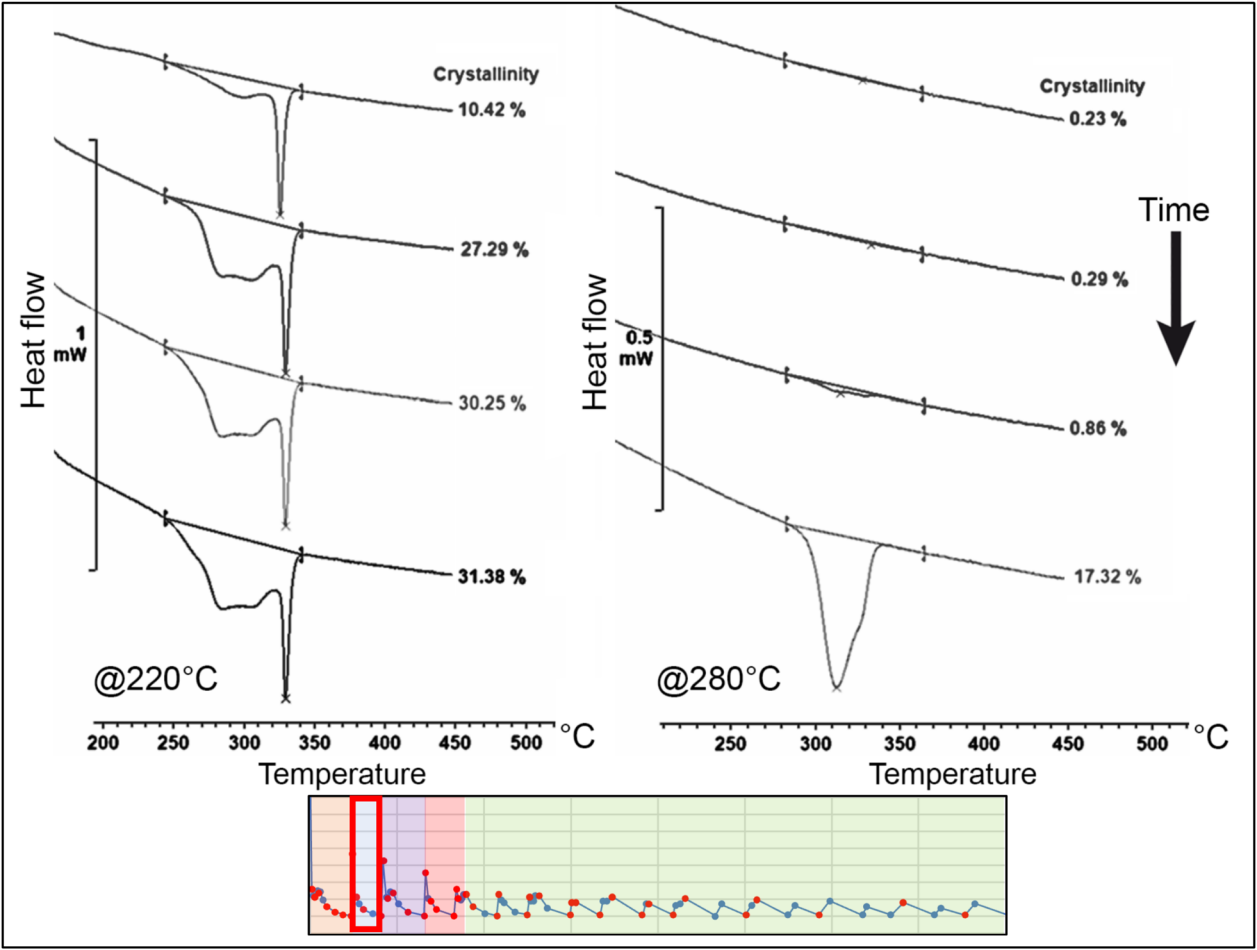
Evolution of the crystallinity between the first and second heat peak (region 2) on node 1 for temperatures of 220 °C and 280 °C^24^.

In the second region, the remelting of the crystalline phase obtained in the first region is evident. Complete melting occurs at a temperature of 280 °C. However, at a temperature of 220 °C, the remelting is only partial. The remaining melting peak appears narrow and constrained on the right side of the original melting range. This suggests a process of reorganization of the crystals that were not melted during the deposition of the subsequent layer, resulting in increased melting temperatures. The melting curves for 220 °C and 280 °C obtained in the second region are illustrated in Fig. 11.

**Figure 12:**
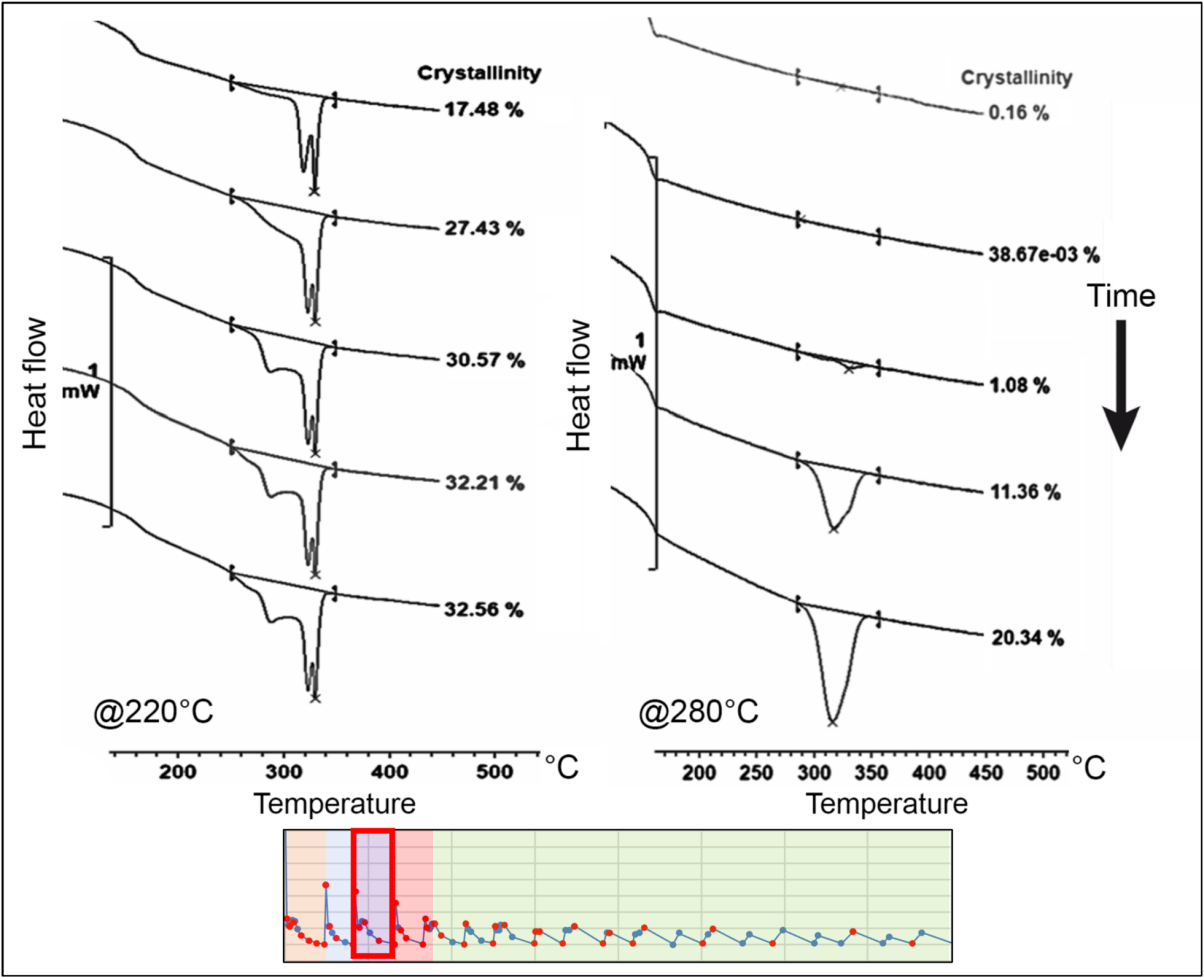
Evolution of the crystallinity between the second and third heat peak (region 3) on node 1 for temperatures of 220 °C and 280 °C^24^.

In the third region, a similar behaviour was observed, and once again, the remelting of the crystalline phase can be assessed. At the higher temperature, complete melting is observed, while at 220 °C, partial remelting occurs. An interesting detail for this region is that the remelting at 220 °C promotes the reorganization of the remaining crystals to a lower temperature level compared to the previous region. This results in the formation of two distinct and narrow peaks concentrated to the right of the temperature range of the original melting peak. The evolution of the melting curves for 220 °C and 280 °C obtained in the third region is depicted in Fig. 12.

In the fourth region, both temperatures exhibit partial remelting of the substrate. However, for the temperature of 280 °C, the remaining crystals are detected in small quantities in the form of a narrow endothermic peak, similar to the behaviour previously observed at 220 °C.

At 220 °C, a third endothermic peak emerges, following the two peaks obtained in the previous regions. This gives rise to a PEEK 450G microstructure with three melting peaks, formed by the material's reorganization during the partial remelting induced by temperature fluctuations in this specific region of the printing process.

**Figure 13:**
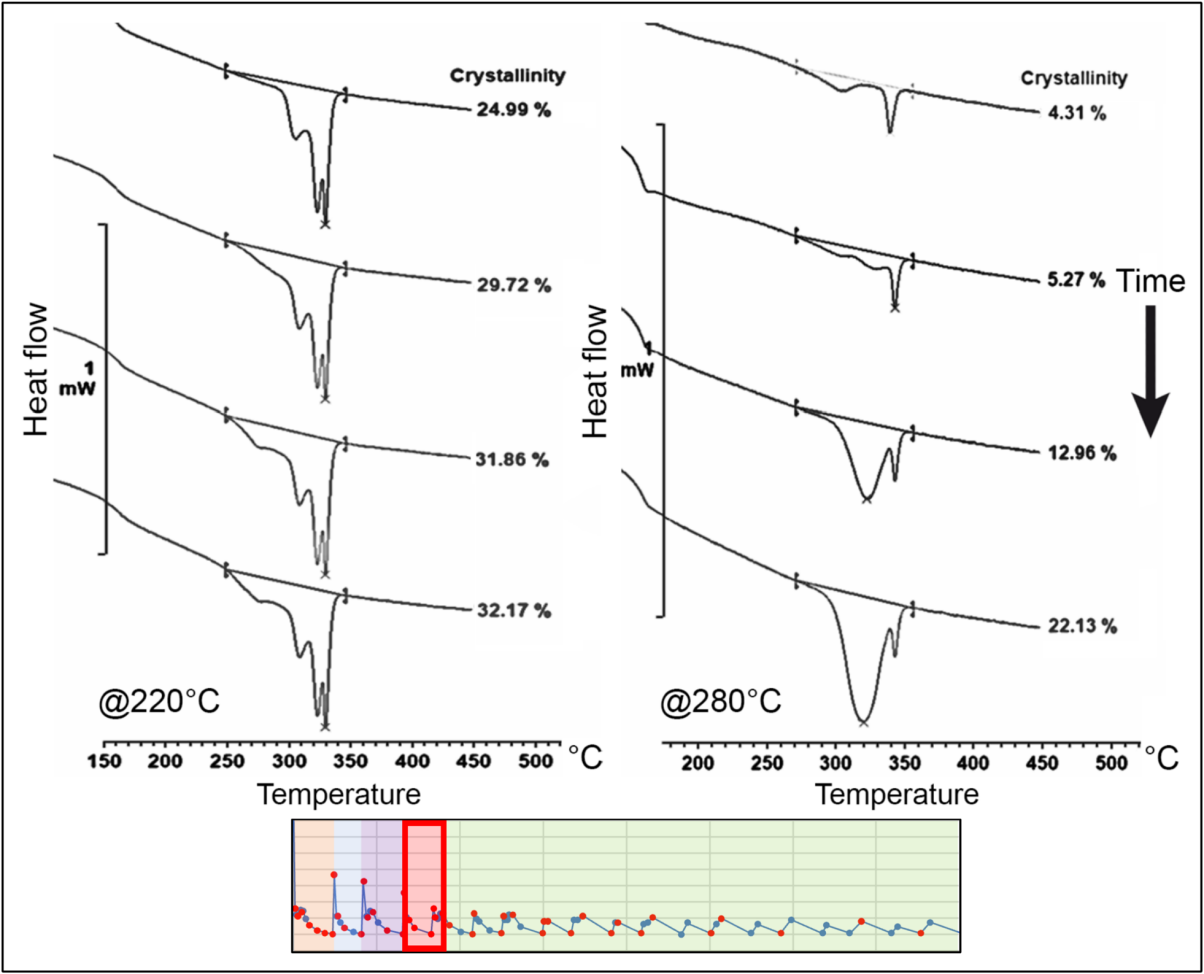
Evolution of the crystallinity between the third and fourth heat peak (region 4) on node 1 for temperatures of 220 °C and 280 °C^24^.

It is noteworthy that this behaviour was not observed for node 2, indicating that different regions of the same printed filament may exhibit distinct morphologies depending on the characteristics of the thermal profile to which they are exposed. The evolution of melting peaks in this region for both temperatures, 220 °C and 280 °C, is illustrated in Fig. 13.

In the fifth and sixth regions, the thermal profile of the process exhibited smaller temperature fluctuations and lacked significant temperature peaks. Consequently, substantial substrate remelting processes were no longer observed. In these regions, crystallinity gradually increased, accompanied by the development of a fourth endothermic peak at a temperature of 220 °C.

It is noteworthy that the shape of the melting curve remained relatively stable throughout the process, maintaining the distinct peaks formed at the beginning of the printing process. For the temperature of 220 °C, the thermogram displayed four distinct peaks, each linked to the thermal effect produced by the deposition of adjacent filaments at the considered node.

**Figure 14:**
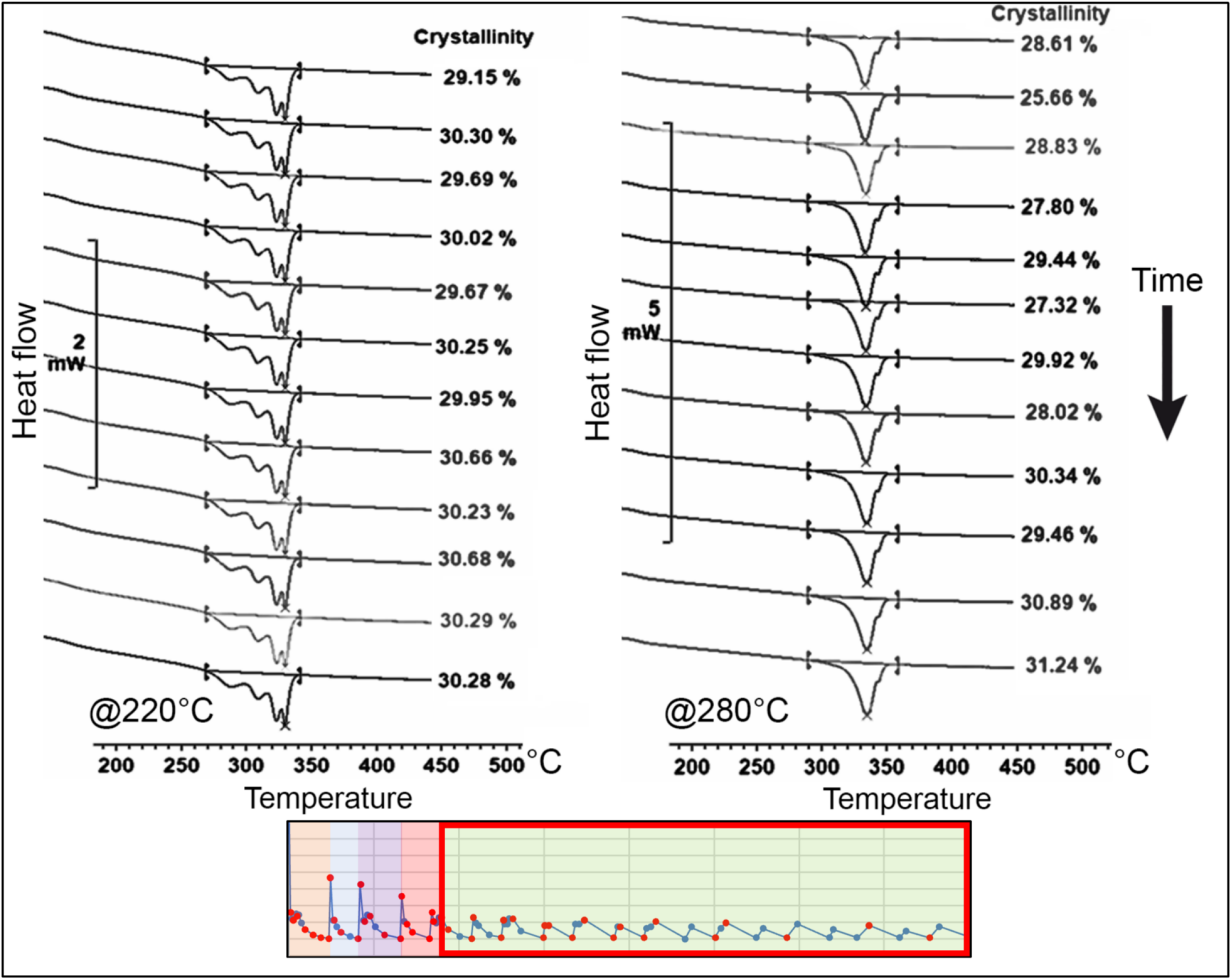
Final evolution of the crystallinity on node 1 for temperatures of 220 °C and 280 °C^24^.

In contrast, for the temperature of 280 °C, a more conventional melting curve was obtained. Although the remaining peak from the partial remelting of the material could still be observed, it was reduced and supressed by the emergence of a new endothermic peak. This peak was formed from the new crystalline phase developed after successive remelting processes. The final evolution of the melting curves for temperatures of 220 °C and 280 °C is depicted in Fig. 14.

The evolution of crystallinity for both tested temperatures provided interesting insights into the process. At lower temperatures, the crystallisation rate was higher. On the other hand, with increasing temperature, there was a delay in the onset of crystallisation. Moreover, higher temperatures tended to induce greater fluctuations in the degree of crystallinity throughout the process.

**Figure 15:**
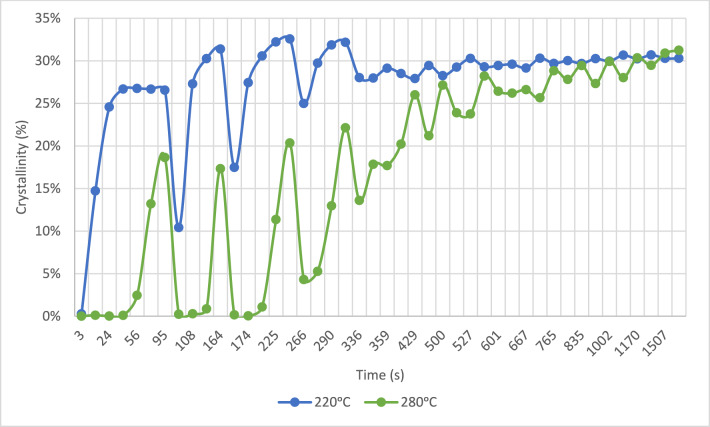
Crystallisation evolution within the MEX process on node 1 for 220 °C and 280 °C^24^.

At higher temperatures, a greater number of complete remelting processes were observed, whereas at lower temperatures, these processes remained incomplete. This disparity significantly influenced the resulting microstructure of the material, presented in the next section. The variation in crystallinity for the two tested temperatures is illustrated in Fig. 15.

It was observed that the higher the temperature reached during the remelting peak, the greater the remelting of the crystalline phase of the build surface. This increased remelting should lead to greater mobility of the polymeric chains, enhancing interlayer entanglement and improving the mechanical properties of the resulting material.

### Morphology analysis

#### Morphology analysis for node 2 with substrate temperature of 220 °C and 280 °C with a return time of 167 s

The sample processed with the 220 °C thermal profile had a mass of 77 ng, while the sample processed using the thermal profile for the substrate temperature of 280 °C had a mass of 265 ng.

**Figure 16:**
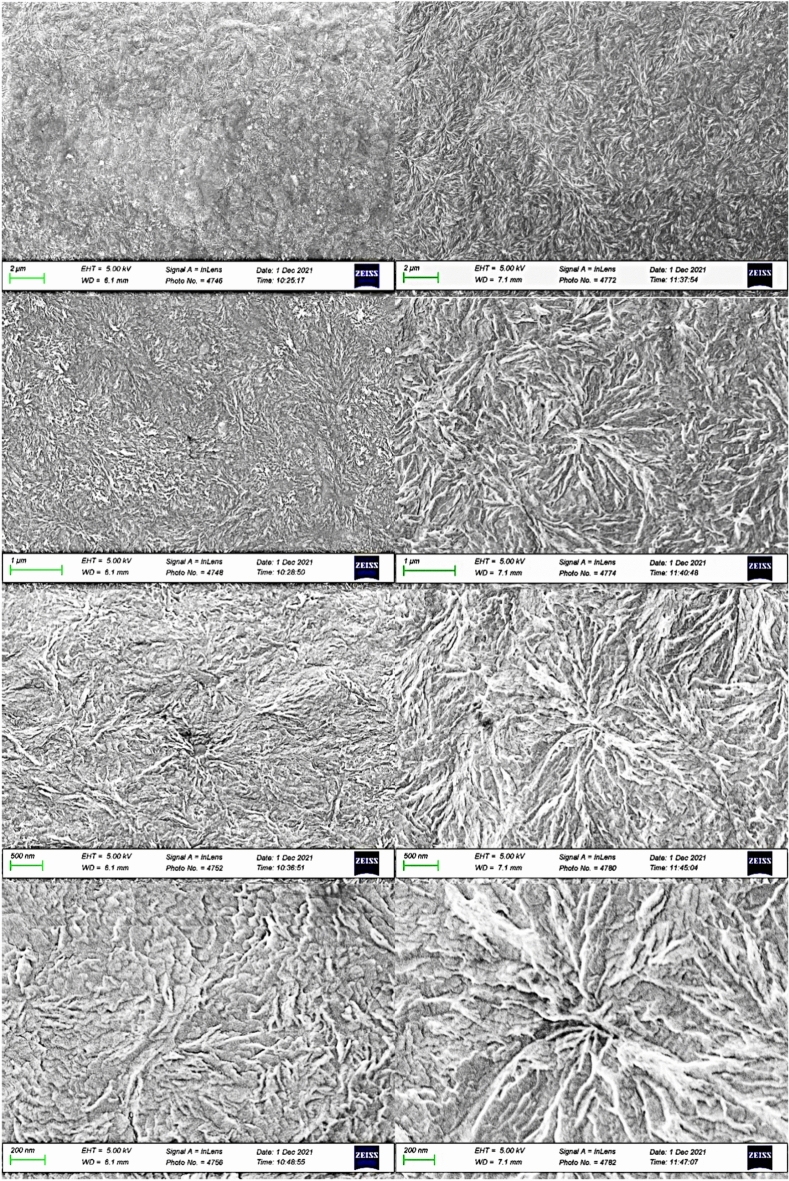
Microstructures on node 2: left: 220 °C, right: 280 °C^24^.

A set of images with increasing magnification for both process configurations is depicted in Fig. 16. Samples on the left were processed with a substrate temperature of 220 °C, while samples on the right were processed at 280 °C. At this initial magnification, the images illustrate the formation of larger spherulites for the material processed with a higher substrate temperature. This crystalline structure, characterized by larger spherulites, results from the increased mobility of the polymeric chains achievable at higher processing temperatures. Consequently, there is a lower density of spherulites with less impingement between them.

Clear differences can be observed between each process configuration, particularly in the higher temperature sample at the highest magnification. Here, a single spherulite covers the entire image, contrasting with the more chaotic microstructure observed in the sample processed at a lower temperature.

#### Morphology for node 1 with substrate temperature of 220 °C and 280 °C and a return time of 97 s

The first sample, weighing 110 ng, was used to simulate the process with a substrate temperature of 220 °C, while the second sample, weighing 95 ng, was used for the temperature of 280 °C.

**Figure 17:**
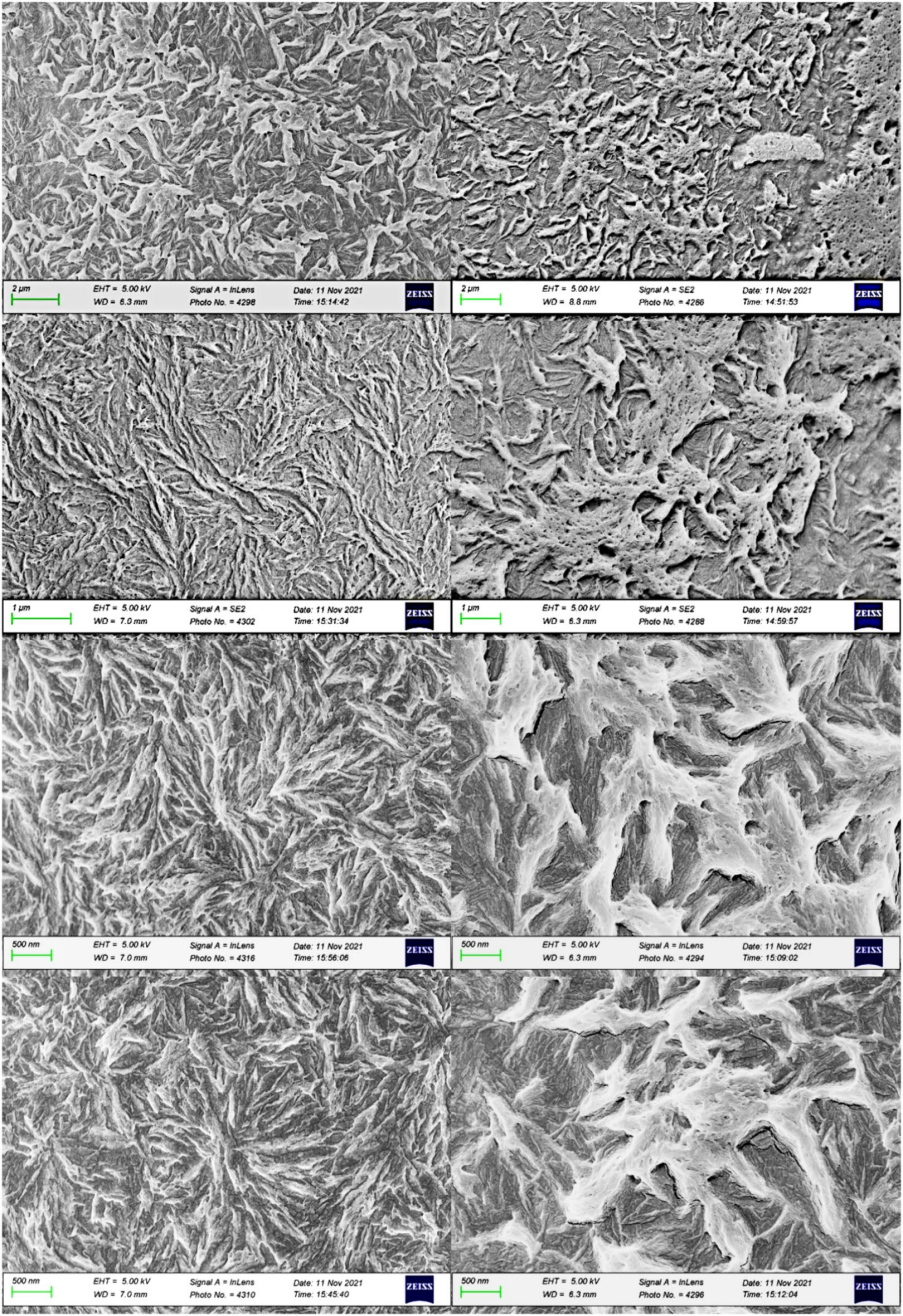
Microstructures for the process simulation on node 1: left: 220 °C, right: 280 °C^24^.

A set of images with increased magnification for the lower and higher temperature samples is shown in Fig. 17. Distinct morphological structures were observed for each substrate temperature used during PEEK processing. In this experiment, however, the greater number of crystalline phase remelting cycles associated with a shorter return time generated a more cohesive structure at 220 °C. In contrast, for 280 °C, crystalline regions organized in clusters and are distributed in a more random manner, presenting structures with less organization and refinement of crystallites.

The differences are noticeable for each process configuration. It's interesting to observe the structural details produced at lower temperatures in this setup, showcasing refined crystallites and lamellae compared to the structure obtained at higher temperatures. This morphology aligns with the simulation of the process at the tangential point of the filament (node 1), where multiple heat inputs from three adjacent filaments result in a greater number of remelting processes and reorganizations of the polymeric chains. This phenomenon explains the presence of larger spherulites compared to the structure previously observed for node 2 at the same temperatures.

Furthermore, the successive reheating processes led to even greater growth of the lamellae, resulting in a microstructure with thick lamellae at the higher temperature (280 °C).

#### Spherulite morphology analysis

**Figure 18:**
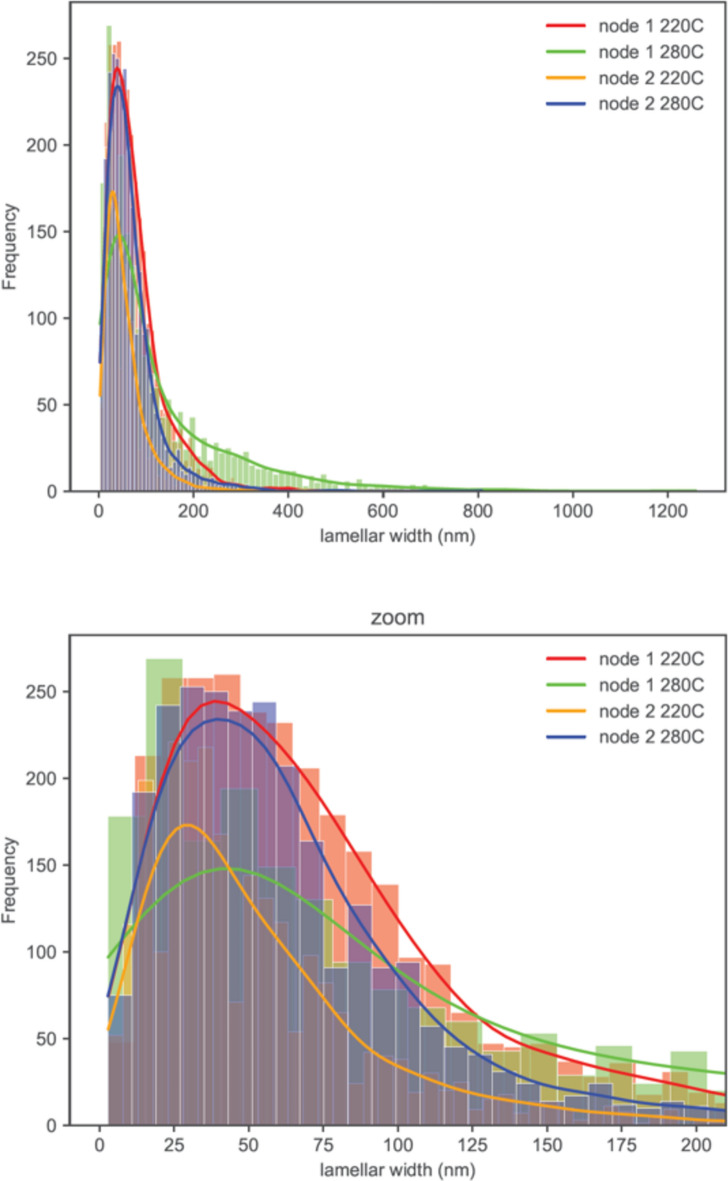
Histogram of observed lamellar thickness distribution with its zoom.

The histogram of lamellar thickness is shown in Fig. 18 with overlaid profile plots, comparing the frequency distributions of measured lamellar thickness at two distinct nodes, each at temperatures of 220 °C and 280 °C. The profiles of Node 1 at 220 °C and Node 2 at 280 °C are similar in shape, both peaking sharply and exhibiting a rapid decline. These distributions suggest a higher frequency of 40 nm lamellar thickness with a rapid decrease in larger lamellar thicknesses. In contrast, the distribution for Node 1 at 280 °C shows a more gradual descent, indicating a broader spread of the measured lamellar thickness, featuring a large number of lamellae thicker than 200 nm.

To further correlate lamellar thickness distribution with crystallinity evolution during the printing process, we applied two crystallinity thresholds, namely 20% and 27%, to Figs. 16 and 17. This analysis determined the time durations within the printing process during which crystallinity either exceeds 27% or falls below 20%. The results are summarized in Table 2.

**Table 2:** Summary of the total time duration at each condition when the crystallinity is above 27% and is below 20% (calculated from Figs. 16 and 17).

Condition	Time duration (s) at crystallinity > 27%	Time duration (s) at crystallinity < 20%	Lamellae size
Node 1, 220 °C	1581	26	Thin
Node 1, 280 °C	1008	349	Thick
Node 2, 220 °C	541	29	Intermediate
Node 2, 280 °C	1441	75	Thin

Node 1 at 220 °C and Node 2 at 280 °C experienced longer time durations during which crystallinity is above 27%. This could result in the formation of numerous thin lamellae, as described by the sharp distribution profiles in Fig. 18. Conversely, Node 1 at 280 °C experienced a longer time duration during which crystallinity falls below 20%, leading to the development of a substantial quantity of thick lamellae, evidenced by the shallow and broad profile in Fig. 18.

#### Morphology for node 1 with substrate temperature of 220 °C during remelting by subsequent filament deposition

**Figure 19:**
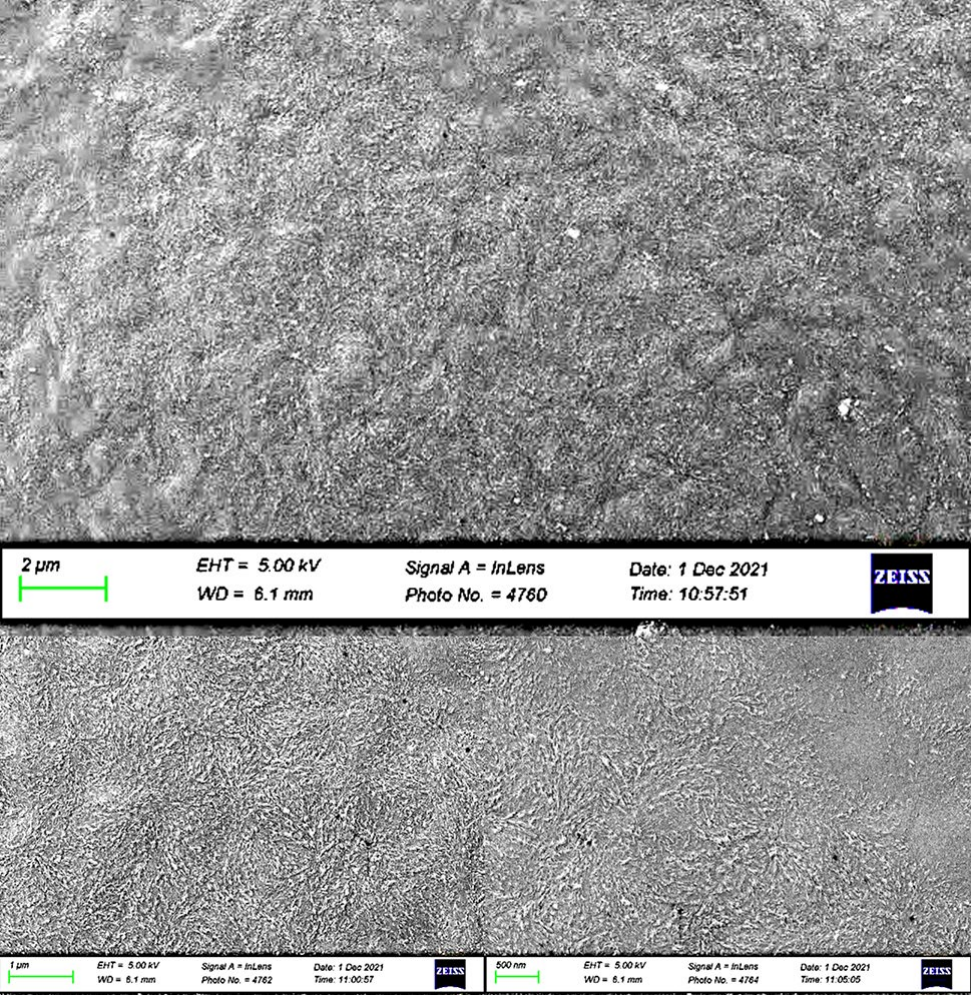
Microstructures for node 1 at 220 °C during the first remelting peak^24^.

This sample, weighing 259 ng, was used to simulate the process with a substrate temperature of 220 °C during the remelting peak. Figure 19 provides increasing magnification on the sample.

The heat input from the subsequent filament reduced the previously observed crystalline structure for this configuration, leading to an increase in amorphous regions with lamellae and evenly distributed crystalline embryos. A higher temperature in the combined building chamber contributes to greater remelting of the crystalline phase present in the substrate, enabling increased entanglement between the polymeric chains in the amorphous form. The microstructure observed during this process exhibits variations in the degree of non-remelted crystalline regions, which vary according to the process characteristics.

## Conclusions

A detailed analysis of the crystallinity evolution and resulting morphology for MEX process was achieved in this study through the examination of melting curves and crystallinity evolution across the entire process thermal profile using FSC and SEM analysis.

The initial stage of the process, from material extrusion to just before the deposition of the subsequent layer, revealed significant differences in crystallinity evolution between temperatures of 220 °C and 280 °C. At 220 °C, rapid crystalline growth occurred, reaching around 20–26% crystallinity before the deposition of the second layer. In contrast, at 280 °C, crystal growth was slower initially, but reached 28% crystallinity at the same critical point.

Further analysis of subsequent regions highlighted distinct behaviours based on temperature variations. At lower temperatures, we observed lower melting temperature peaks, indicating faster crystallisation rates and reduced polymer chain reorganization. In contrast, higher temperatures delayed crystallisation onset, resulting in greater reorganization of crystals and higher melting point peaks.

Notably, the analysis of different regions on node 1 provided additional insights into the influence of adjacent filament heat input on crystallinity evolution. Processes such as partial remelting, crystal reorganization, and the development of distinct melting peaks, impacting the microstructure of the material, were observed.

The microstructure of PEEK was successfully observed by SEM directly from FSC chip samples. The influence of the building substrate temperature on the morphology of PEEK is clear in the obtained images. Higher temperatures promote the formation of larger spherulites and result in crystalline regions with thick and well-developed lamellae, in contrast to the microstructure obtained at lower temperatures.

When comparing the evaluated nodes, it is noticeable that the presence of multiple polymer remelting processes, particularly in node 1, enhances the formation of a structure with more developed and thick lamellae. This is a result of increased chain mobility stimulated by each temperature peak during the processing at this node.

Evaluation of the remelting peak region reveals a microstructure consisting mostly of amorphous regions, with uniformly distributed spherulite embryos within the material. This is in line with expectations after experiencing melting during this specific stage of the process.

The lamellar thickness distribution analysis showed that higher temperatures result in thinner lamellae. High crystallinity (above 27%) correlates with thinner lamellae, while low crystallinity (below 20%) leads to thicker lamellae. Longer durations of high crystallinity produce thinner lamellae, whereas longer durations of low crystallinity result in thicker ones. These findings suggest that optimizing printing parameters can influence crystallinity and lamellar characteristics, providing insights for additive manufacturing process optimization.

The combination of SEM and FSC techniques has proven to be a powerful tool for assessing the microstructure of polymers subjected to complex thermal profiles, enabling the in-situ observation of microstructural evolution during thermal processing. The methodology used can provide new insights into the dynamic processes that govern crystallisation in semi-crystalline polymers like PEEK. Future research could further explore the potential of this approach to study other polymers and composite materials, offering a broader understanding of the relationship between processing conditions, microstructure, and material properties.

## Data Availability

The datasets used and/or analysed during the current study are available from the corresponding authors on reasonable request.
